# Comparison of Old and New Systemic Treatments for Moderate to Severe Atopic Dermatitis

**DOI:** 10.1007/s11882-024-01145-x

**Published:** 2024-04-18

**Authors:** Hyun J. Yim, Tiffany Jean, Peck Y. Ong

**Affiliations:** 1 Department of Internal Medicine, Kaiser Los Angeles Medical Center, Los Angeles, CA USA; 2 Department of Allergy, Asthma, & Clinical Immunology, Kaiser Los Angeles Medical Center, Los Angeles, CA USA; 3 Division of Clinical Immunology and Allergy, Children’s Hospital Los Angeles, University of Southern California,, 4650 Sunset Blvd., MS #75, Los Angeles, CA 90027 USA

**Keywords:** Eczema, Biologics, Immunosuppressants, JAK inhibitors, Treatment options

## Abstract

**Purpose of Review:**

Historically, systemic treatments for atopic dermatitis (AD) primarily consisted of immunosuppressive agents such as corticosteroids and Disease Modifying Antirheumatic Drugs (DMARDS), which provided symptomatic relief but often had long-term adverse effects. Newer treatments have shown significant efficacy with less side effects in clinical trials. This review discusses and compares conventional and newer systemic treatments for AD.

**Recent Findings:**

Newer medications for AD including dupilumab, tralokinumab, lebrikizumab, and oral JAK inhibitors have been shown to be safe and efficacious. High dose cyclosporine and dupilumab were more effective than methotrexate and azathioprine in improving clinical signs of AD. High-dose upadacitinib was shown in another meta-analysis to be most effective in the measured outcomes but had the highest frequency of adverse events.

**Summary:**

Targeted biologic treatments are increasingly favored over traditional immunosuppressive treatments of AD. Treatment can be individualized based on potency, adverse side effects, mechanism of action, and administration preference. Ongoing research continues to expand treatment options for AD.

## Introduction

Atopic Dermatitis (AD) is a chronic inflammatory skin disorder that affects 7% of adults and up to 20% of children across the world [1]. It often presents in childhood and can persist into adulthood. The presentation commonly involves skin pruritus, dryness, papules and erythema. Persistent scratching leads to crusting and lichenification of the skin [2]. The pathogenesis of AD involves skin barrier dysfunction from filaggrin and lipid deficiency, genetic predisposition, environmental factors, and a predominantly Th2 immune dysregulation [3].

After diagnosis of AD, it is useful in both clinical and research settings to grade the severity of AD for treatment and for monitoring response to treatments. Scoring tools such as the Eczema Area and Severity Index (EASI) assesses the severity of redness, thickness, excoriation, and lichenification, and the percentage of skin involvement in the head, trunk, arms, and legs. EASI-75 indicates an improvement of 75% from EASI baseline. The Investigator Global Assessment (IGA) is used to grade AD based on morphological description, with 0 being “clear”, 1 “almost clear”, 2 “mild, 3 “moderate”, and 4 “severe”. It is also referred to as investigator static global assessment (ISGA), or validated IGA(vIGA). The Numerical Rating Scale (NRS) is a rating scale 0–10 of itch with 0 being no itch and 10 being severe itch. An improvement of itch by 4 points or more (NRS-4) is considered significant. Quality of Life (QoL) is measured by Dermatology Life Quality Index (DLQI), or Children’s DLQI (CDLQI), and consists of ten questions about how AD affects QoL. The score ranges from 0 to 30, with 30 being most impaired QoL.

## Conventional Management and Treatments

General management of AD includes skin hydration with emollients, and avoiding triggers such as excessive dryness, skin irritants, infection and environmental allergens [4]. When these interventions are not enough to manage symptoms, topical corticosteroids (TCS) or topical calcineurin inhibitor (TCI) may be used.

Topical corticosteroids (TCS) have been the mainstay of treatment for AD [5]. TCS works by decreasing inflammation and can be used for active flares and for maintenance therapy [6]. They are categorized by potency, from class I being the most potent to class VII being least potent [7]. Adverse effects (AE) are uncommon but can include skin atrophy, telangiectasia, striae, and acneiform eruptions [8]. Systemic AE such as adrenal insufficiency are rare with low-to-moderate potency (potency 4–7) TCS for a short-term period (2–4 weeks) [9]. TCS are effective in most patients and are generally safe when used intermittently to treat eczema flares [10•]. However, some patients fear steroids due to the possible AE and nonadherence is common. Additionally, TCS may be insufficient for some patients with moderate-to-severe AD [11]. In those cases, tacrolimus ointment, which is a topical calcineurin inhibitor (TCI), can be used. Tacrolimus inhibits T-lymphocyte activation, and is FDA approved for moderate-to severe AD [12]. It can be used on relapsing AD and on sensitive skin areas. Tacrolimus 0.1% efficacy is similar to TCS class IV, and it has been demonstrated to have a greater improvement in the EASI compared to moderate potency TCS class V [13, 14••] The main AE consists of local burning and stinging. Although tacrolimus carries a black box warning of cancer risk, studies during the past 20 years and a recent meta-analysis showed that TCI do not increase the risk of cancer [15, 16, 17•]. Pimecrolimus is another topical calcineurin inhibitor, but it is indicated for mild-to-moderate AD, and has a potency between TCS classes V- VI [14••, 18].

Ruxolitinib 1.5% cream is a newer topical Janus kinase inhibitor that has been approved for mild-to-moderate AD in 2021. It has not been part of the conventional treatment armamentarium extensively, therefore, it will be discussed in more detail in the chapter on topical treatments.

Before the approval of the first biologic for AD (Dupilumab), the only FDA-approved systemic treatment for AD was corticosteroids. Corticosteroids have a quick onset of action and have been used in severe, acute episodes of AD. However, they are not appropriate for maintenance therapy because of its AE including rebound flares, and they should be avoided in patients younger than 12 years old due to AE on growth [19]. Given these restraints, systemic immunosuppressants, or disease modifying antirheumatic drugs (DMARDs), were often used for those who had failed topical treatments for AD. These DMARDs include cyclosporine, methotrexate, azathioprine, and mycophenolate mofetil. Among these, cyclosporine is the only DMARD that is approved for AD in the European Union.

### Disease Modifying Antirheumatic Drugs

Cyclosporine is a calcineurin inhibitor that blocks transcription of interleukin-2 (IL-2) and cytokines and decreases activation of T lymphocytes [20]. It works quickly (within 2–3 weeks) but is not recommended for use over one year [21]. AE include nephrotoxicity, hypertension, increased risk of malignancy, increased risk of infections, gastrointestinal upset, and lab abnormalities. While it was shown to be effective in adults and children, it was better tolerated in children [22].

Methotrexate (MTX) is a folic acid antagonist that works by inhibiting dihydrofolate reductase, which inhibits cell division and lymphocyte proliferation and consequently decreases inflammation. It is given orally, and the effects are seen after a minimum of 6 weeks of treatment. Folate helps reduce MTX toxicity, which can cause nausea, vomiting, diarrhea, or fatigue. Rare but serious AE include hepatoxicity and pancytopenia, which requires laboratory monitoring [21]. Although MTX takes longer to work, it can be continued as maintenance therapy as it has been shown to be efficacious and safe for longer-term control of AD [23, 24].

Azathioprine is a 6-mercaptopurine analog that works by inhibiting purine synthesis and by decreasing inflammatory response. It has slower onset of action (weeks to months) [25]. AE include gastrointestinal upset, increased risk of infection and malignancy, bone marrow suppression, liver toxicity, and rare hypersensitivity syndrome [21]. Azathioprine requires laboratory monitoring for bone marrow suppression and liver toxicity. There are only few observational studies on children with AD [26, 27].

Mycophenolate mofetil binds inosine monophosphate dehydrogenase and inhibits purine synthesis, which inhibits lymphocyte activation. It has a relatively slow onset of action (6–8 weeks) and is best for maintenance therapy. AE include headaches, herpes infection, gastrointestinal upset, risk of infection, liver function test abnormalities, myelosuppression, and risk of carcinogenesis. It was shown to have good efficacy and safety in pediatric AD [28, 29], with a systemic review and meta-analysis showing 77.6% having partial or full remission of AD symptoms by SCORAD scores [30].

Although MTX, azathioprine, and mycophenolate have shown efficacy over placebo, the most recent JTF Practice parameters recommend against the use of these medications for AD.

## Biologics

### Dupilumab

Dupilumab is a monoclonal antibody that blocks the IL-4 receptor alpha, resulting in inhibition of IL-4 and IL-13 [31••] (Table 1). It was approved by Food and Drug Administration (FDA) for adults with moderate-to-severe AD in 2017, making it the first biologic approved for AD. A randomized, double-blinded, placebo-controlled (RDBPC), phase 3 study (LIBERTY AD CHRONOS), showed that adults who received dupilumab 300mg/TCS qw and dupilumab 300mg/TCS q2w had greater achievements in endpoints of IGA 0/1, EASI-75, NRS-4, and mean DLQI change from baseline than placebo at week 16 and week 52 [32••] (Table 2).

**Table 1:** Summary of new systemic treatments for moderate-to-severe AD

**Treatment**	**Mechanism of Action**	**Route**	**FDA approval**	**Adverse Events**
Dupilumab	Monoclonal antibody that blocks IL-4 receptor alpha, inhibiting IL-4 and IL-13	Subcutaneous injection	Yes, for > 6 months old	- Injection site reactions/pain - Conjunctivitis - Nasopharyngitis - URI - Sinusitis - Facial erythema - Eosinophilia
Tralokinumab	Monoclonal antibody that binds to and inhibits IL-13	Subcutaneous injection	Yes, for ≥ 12 years old	- Injection site reactions - Conjunctivitis - URI - Headache - Eosinophilia
Lebrikizumab	Monoclonal antibody that binds to and inhibits IL-13	Subcutaneous injection	No; in reviewal for approval in Europe	- Conjunctivitis - Nasopharyngitis - Injection site reactions - Eosinophilia
Upadacitinib	JAK-1 inhibitor	Oral	Yes, for ≥ 12 years old	- Acne - Herpes zoster, herpes simplex - Nasopharyngitis - URI - Elevation of creatine phosphokinase (CPK) levels - Black box warning of malignancy, cardiovascular disease, emboli, and serious infections
Abrocitinib	JAK-1 inhibitor	Oral	Yes, for ≥ 12 years old	- Nausea - Acne - Herpes zoster, herpes simplex - Nasopharyngitis - Headache - URI - Elevation of CPK levels - Black box warning of malignancy, cardiovascular disease, emboli, and serious infections
Baricitinib	JAK-1 and JAK-2 inhibitor	Oral	No; approved by European medicines agency for ≥ 18 years old	- Nasopharyngitis - Herpes zoster, simplex virus - Headache - Abdominal pain - Back pain - Conjunctivitis

**Table 2:** Efficacy of systemic immunomodulators for moderate-to-severe AD

Treatment	Study participants	No. of participants	Dosing	% subjects achieving IGA 0/1	% subjects achieving EASI-75	% subjects achieving NRS-4	Improvement of QoL from baseline (DLQI or CDLQI)
Dupilumab	Liberty AD Chronos Adults Wk 16	740	Placebo	12	23	20	-5.3
			300mg qw	39	64	51	-10.5
			300mgq2w	39	69	59	-9.7
	Wk 52		Placebo	13	22	13	-5.6
			300mg qw	40	64	39	-10.7
			300mgq2w	36	65	51	-10.9
	12–17 y.o* Wk 16	251	Placebo	2.4	8.2	4.8	-5.1
			300mg q4w	17.9	38.1	26.5	-8.8
			300mgq2w	24.4	41.5	36.6	-8.5
	6–11 y.o** Wk 16	367	Placebo/TCS	11.4	26.8	12.3	-6.4
			300mg q4w/TCS	32.8	69.7	50.8	-10.6
			300mg q2w/TCS	29.5	67.2	58.3	-10.7
	6 mo- < 6 y.o*** Wk 16	162	Placebo/TCS	4	11	9	-2.5
			200-300mg/TCS	28	53	48	-10
Tralokinumab	ECZTRA Adults Wk 16	380	Placebo/TCS q2w	26.2	35.7	34.1	-8.9
			300mg/TCS q2w	38.9	56	45.4	-11.7
	Wk 32		Pooled 300mg q2w/q4w	48.4	70.2		-13.7/-14.2
Lebrikizumab	ADvocate 1 > 12 y.o Wk 16	424	Placebo q2w	12.7	16.2	13	-4.4
			250mg q2w	43.1	58.8	45.9	-9.9
	ADvocate 2 > 12 y.o Wk 16	427	Placebo	10.8	18.1	11.5	-5.0
			250mg q2w	33.2	52.1	39.8	-9.4
Upadacitinib	AD Up > 12 y.o Wk 16	901	Placebo qd	28.5	26	15	
			15mg qd	40	65	51.7	
			30mg qd	59	77	63.9	
	Wk 52		15mg qd	33.5	50.8	45.3	
			30mg qd	45.2	69	57.5	
Abrocitinib	JADE MONO-1 > 12 y.o Wk 12	387	Placebo	8	12	15	-4.2
			100mg qd	24	40	38	-7
			200mg qd	44	63	57	-9.1
	JADE MONO-2 > 12 y.o Wk 12		Placebo	9.1	10.4	11.5	DLQI/CDLQI -3.9/-2.7
			100mg qd	28.4	44.5	45.2	-8.3/-4.8
			200mg qd	38.1	61	55.3	-9.8/-9.7
	JADE COMPARE > 18 Y/O Wk2		Placebo/TCS			13.8	
			100mg/TCS qd			31.8	
			200mg/TCS qd			49.1	
			Dupilumab 300mg/TCS q2w			26.4	
	Wk 12		Placebo/TCS	14	27.1		
			100mg/TCS qd	36.6	58.7		
			200mg/TCS qd	48.4	70.3		
			Dupilumab 300mg/TCS q2w	36.5	58.1		
	Wk 16		Placebo/TCS	12.9	30.6		
			100mg/TCS qd	34.8	60.3		
			200mg/TCS qd	47.5	71		
			Dupilumab 300mg/TCS q2w	38.8	65.5		
	JADE TEEN 12–17 y.o Wk 12		Placebo/TCS	24.5	41.5	29.8	
			100mg/TCS qd	41.6	68.5	52.6	
			200mg/TCS qd	48.2	72	55.4	
	JADE EXTEND Wk 48	1116	100mg qd	39	67	51	
			200mg qd	52	82	68	
Baricitinib	BREEZE-AD4 Adults Wk 16	463	Placebo/TCS qd	10	17	8	-4.95
			1mg/TCS qd	13	23	23	-6.18
			2mg/TCS qd	15	n/a	23	-6.57
			4mg/TCS qd	22	32	38	-7.95
	Wk 52		Placebo/TCS qd	16	27	19	-4.76
			1mg/TCS qd	20	33	31	-6.41
			2mg/TCS qd	18	30	23	-6.79
			4mg/TCS qd	23	10.1	34	-7.02
	BREEZE-AD PEDS 2 yo- < 18 yo Wk 16	489	Placebo	16.4	32	16.4	
			1mg/TCS qd	18.2	32.2	17.5	
			2mg/TCS qd	25.8	40	25.8	
			4mg/TCS qd	41.7	52.5	35.5	

*For the study, subjects weighing < 60kg received Dupilumab 200mg and ≥ 60kg received Dupilumab 300mg; **For the study, subjects weighing 15 to < 30kg received Dupilumab 100mg and ≥ 30kg received Dupilumab 200mg; ***For the study, subjects weighing ≥ 5 kg to < 15 kg received Dupilumab 200 mg; subjects ≥ 15 kg to < 30 kg received Dupilumab 300 mg

A 16-week (RDBPC) study randomized adolescents aged 12–17 years old to dupilumab 300mg q4w, and dupilumab q2w [33]. The proportion of participants achieving the endpoints are as follows for placebo, dupilumab q4w, and dupilumab q2w, respectively: IGA 0/1 2.4%, 17.9%, 24.4%; EASI-75 8.2%, 38.1%, 41.5%; NRS-4 4.8%, 26.5%, 36.6%; improvement of CDLQI scores from the baseline -5.1, -8.8, -8.5 (Table 2). Dupilumab q2w was shown to be more efficacious when compared to placebo and dupilumab q4w (Table 2).

A 16-week (RDBPC) phase 3 trial randomized participants aged 6–11 years old into placebo/TCS vs. dupilumab 300mg/TCS q4w/TCS vs. dupilumab/TCS q2w [34]. The proportion of participants achieving the endpoints are as follows for placebo/TCS, dupilumab/TCS q4w, and dupilumab/TCS q2w, respectively: IGA 0/1 11.4%, 32.8%, 29.5%; EASI-75 26.8%, 69.7%, 67.2%; NRS-4 12.3%, 50.8%, 58.3%; improvement of CDLQI -6.4, -10.6, -10.7 (Table 2).

A phase 3 (RDBPC) trial was also carried out in children 6 months to 6 years old with moderate-to-severe AD (IGA 3–4) [35]. Participants were randomized into placebo/TCS vs. dupilumab/TCS q4w for 16 weeks. The proportion of participants achieving the endpoints are as follows for placebo/TCS, dupilumab/TCS q4w, respectively: IGA 0/1 4%, 28%; EASI-75 11%, 53%; NRS-4 9%, 48%, improvement of CDLQI -2.5, -10 (Table 2). Overall, dupilumab significantly improved AD severity, itch, and QoL, and was approved in 2022 for patients ≥ 6 months old with moderate-to-severe AD.

AE in children and adults from dupilumab include nasopharyngitis, upper respiratory infection (URI), sinusitis, herpes infection, conjunctivitis, and injection site reactions [32••, 34]. The dupilumab/TCS groups had higher rates of injection-site reactions and conjunctivitis when compared to placebo. Most injection site reactions and conjunctivitis were mild to moderate. Injection site reactions declined over time and conjunctivitis resolved with topical eye treatments. Only one patient in the adult study discontinued due to conjunctivitis. Similar proportions of patients in the treatment groups reported herpes viral infections, but there were lower rates of skin infections, asthma, and allergic rhinitis in the dupilumab groups. Dupilumab is currently also indicated in treatment of asthma, eosinophilic esophagitis, and chronic rhinosinusitis with nasal polyps [36–38].

### Tralokinumab

Tralokinumab is a human monoclonal antibody that binds to and prevents action of IL-13. It is FDA-approved for patients ≥ 12 years old with moderate-to-severe AD. A RDBPC 3 trial (ECZTRA 3) randomized patients 2:1 to subcutaneous tralokinumab 300mg or placebo q2w with TCS as needed for an initial 16 weeks [39]. At week 16, endpoints were achieved by participants in Tralokinumab/TCS q2w and placebo/TCS were as follows respectively: IGA 0/1 38.9%, 26.2%**;** EASI-75 56.0%, 35.7%; NRS-4 45.4%, 34.1%; mean change DLQI change from baseline -11.7, -8.9 (p < 0.001). Overall, of the patients who received tralokinumab/TCS q2w, 53.4% achieved a clinically meaningful improvement in AD.

At week 16, 233 patients who achieved the clinical response criteria (IGA 0/1 and/or EASI-75) with tralokinumab were re-randomized 1:1 to tralokinumab 300mg/TCS q2w or q4w for another 16 weeks [40]. At week 32, % of participants in the pooled tralokinumab q2w and q4w patient groups who achieved the endpoints were as follows: IGA 0/1 48.8%, EASI-75 70.2%. There was no significant difference in efficacy between those receiving tralokinumab q2w vs q4w at week 32.

Most common AE reported ≥ 5% URI, conjunctivitis, headache, and injection‐site reactions. Conjunctivitis was reported more frequently with tralokinumab than placebo in the initial treatment period but were mild or moderate in severity and usually recovered by the end of the initial treatment period, with one patient discontinuing tralokinumab due to conjunctivitis. While more patients treated with tralokinumab had increased eosinophil levels during the initial treatment period, the safety profile of patients with increased eosinophil counts was not significantly different to the overall trial population.

### Lebrikizumab

Lebrikizumab is a monoclonal antibody that binds to IL-13. It is currently in review for approval in Europe for AD but is not yet FDA-approved in the United States. It was studied in two identically designed, (RDBPC), phase 3 trials (ADvocate 1 and ADvocate 2), where participants 12 years and older (weighing ≥ 40kg) were randomly assigned in a 2:1 ratio to receive either subcutaneous lebrikizumab 250mg q2w (loading dose of 500mg at baseline and week 2) or placebo q2w for 52 weeks [41]. At week 16 of ADvocate 1, the lebrikizumab and placebo group respectively achieved IGA 0/1 by 43.1%, 12.7%; EASI-75 by 58.8%, 16.2%; and NRS-4 by 45.9%, 13%. At week 16 of ADvocate 2, lebrikizumab and placebo respectively achieved IGA 0/1 33.2%, 10.8%; EASI-75 52.1%, 18.1%; and NRS -4 by 39.8%, 11.5%.

Patients who responded to lebrikizumab 250 mg q2w at the end of the 16-week induction period were randomized 2:2:1 to receive lebrikizumab q2w, lebrikizumab q4w or placebo q2w for 36 additional weeks [42]. In combining the results of ADvocate1 and ADvocate2 at week 52, the proportion of participants who were able to maintain the endpoints were as follows for lebrikizumab q2w, lebrikizumab q4w and placebo, respectively: IGA 0/1 with a ≥ 2-point improvement 71.2%, 76.9%, 47.9%; EASI-75 78.4%, 81.7%, 66.4%; NRS-4 84.6%, 84.7%, 66.3%. This study demonstrated continued efficacy of lebrikizumab up to week 52, and that an induction period of lebrikizumab Q2W followed by Q4W dosing of lebrikizumab can be sufficient to sustain response for moderate-to-severe AD up to week 52.

The most common AE reported ≥ 5% during both studies for 52 weeks were mild to moderate in severity and included conjunctivitis, nasopharyngitis, AD, and herpes viral infections. There were low frequency of injection site reactions and eosinophilia, and no eosinophil-related disorders were reported.

## Oral Jak Inhibitors

### Upadacitinib

Upadacitinib is an oral Janus kinase (JAK)-1 inhibitor. It is FDA-approved for patients aged 12 years and older with moderate-to-severe AD. A (RDBPC), phase 3 trial (AD Up) randomly assigned participants ≥ 12 years old (1:1:1) to receive upadacitinib 15 mg, upadacitinib 30 mg, or placebo once daily with TCS for 16 weeks [43]. The proportion of patients who achieved the endpoints at week 16 were as follows for upadacitinib 30mg/TCS, upadacitinib 15mg/TCS, and placebo/TCS groups**,** respectively: EASI-75 77%, 65%, 26%; vIGA-AD 0/1 59%, 40%, 28.5%; ≥ 4-point improvement in worst pruritus numerical rating scale (WP-NRS-4) 51,7%, 63.9%, 15%. Significant improvement in itch and severity was observed in treated groups as early as 1 to 2 weeks, as compared to the placebo group.

AD Up is in the ongoing blinded extension (BE) period, and data so far shows that efficacy is maintained through week 52 [44]. At week 52, the proportion of patients who achieved endpoints for upadacitinib 15mg/TCS and upadacitinib 30mg/TCS respectively was: EASI-75 50.8% and 69%; vIGA-AD 0/1 33.5% and 45.2%; WP-NRS- 4 45.3% and 57.5% (Table 2).

AE reported ≥ 10% in either treatment group for 52 weeks included acne (14% in upadacitinib 15mg vs. 18.6% in upadacitinib 30mg), nasopharyngitis, URI, elevation of blood creatine phosphokinase levels, and AD. Rates of serious infections were similar between treatment groups. There were more herpes zoster infections and acne in upadacitinib 30mg vs 15mg (5 vs 4/100 patient years). There was one case of tuberculosis in each 15mg and 30mg group. Other than herpes zoster, most other cases of opportunistic infections were from eczema herpeticum (Kaposi varicelliform eruption) but were nonserious. There were two major adverse cardiovascular events and one venous thromboembolic event but they were deemed unrelated to upadacitinib. There were no reports of gastrointestinal perforation, or lymphoma.

### Abrocitinib

Abrocitinib is an oral JAK-1 inhibitor FDA approved for patients ≥ 12 years old with moderate-to-severe AD. Once daily Abrocitinib as monotherapy was shown to be effective in phase 3 studies of patients ≥ 12 years old in JADE MONO-1 and JADE MONO-2 [45]. JADE MONO-1 randomized participants 2:2:1 to oral abrocitinib 100mg, abrocitinib 200mg, or placebo qd for 12 weeks [46]. The proportion of participants who achieved the primary endpoints from the abrocitinib 100mg qd, abrocitinib 200mg qd, and placebo, were respectively: IGA 0/1 24%, 44%, 8%; EASI-75 40%, 63%, 12%. Secondary endpoints were: NRS-4 38%, 57%, 15%; change in DLQI -7, -9.1, -4.2; and change in CDLQI -6.4, -7.5, -3.9. JADE MONO-2 is a replicate phase 3 trial of JADE MONO-1 with similar results (Table 2). In both trials, anti-itch effect and efficacy were observed in the abrocitinib groups as early as 2 weeks, as compared to the placebo group.

Phase 3 JADE EXTEND is an ongoing study of abrocitinib in participants ≥ 12 years old who completed full treatment period of abrocitinib or placebo in JADE MONO-1, JADE MONO-2, or JADE COMPARE [47]. In JADE EXTEND, participants were randomized to abrocitinib 200mg or 100mg. At week 48, the proportion of patients receiving abrocitinib 200mg and 100mg who achieved IGA 0/1 was 52% and 39%, EASI-75 was 82% and 67%, and peak-pruritus (PP)-NRS-4 was 68% and 51%, respectively. This showed that long-term treatment with abrocitinib had sustained and clinically meaningful improvement in symptoms of AD.

AE that occurred ≥ 5% included nausea, nasopharyngitis, headache, URI, AD. Nausea and URI were the most frequent. Nausea occurred more frequently in the 200mg vs 100mg group (15% vs 6%). Acne and herpes zoster were also reported more frequently in the 200mg vs 100mg group. Serious AE were reported in 3% of abrocitinib 100mg group and 5% in abrocitinib 200mg group, with only 2 events considered to be treatment related (inflammatory bowel disease and acute pancreatitis) [48]. No cases of venous thromboembolism, malignancies, major adverse cardiovascular events, or deaths were observed.

### Baricitinib

Baricitinib is an oral JAK1 and JAK2 inhibitor approved in Europe by the European medicines agency for adults ≥ 18 years old but is not yet FDA approved for AD in the United States. A (RDBPC), phase 3 study (BREEZE-AD4) randomized participants with moderate-to-severe AD 1:1:2:1 to placebo, baricitinib 1mg, 2mg, or 4mg daily with TCS for 52 weeks [49]. Those who achieved EASI-75 at week 16 (primary endpoint) for baricitinib 1mg, 2mg, 4mg, and placebo, respectively, were: 23%, 28%, 32%, 17% (Table 2). While baricitinib 4 mg/TCS was superior to placebo/TCS for EASI 75, baricitinib 1mg/TCS was not superior to placebo for EASI 75. vIGA-AD 0/1 was achieved by 13%, 15%, 22%, placebo 10%; NRS-4 23%, 23%, 38%, placebo 8%. Baricitinib at all studied doses were superior to placebo in achieving NRS-4. DLQI, LS mean was as follows: -6.18, -6.57, -7.95, -4.95, with baricitinib 4mg showing significantly greater improvement in DLQI than placebo.

At week 52, results were EASI-75 33%, 30%, 10.1%, 27%; vIGA-AD 0/1 20%, 18%, 23%, 16%; NRS-4 31%, 23%, 34%, 19%; DLQI -6.41, -6.79, -7.02, -4.76. EASI-75 and vIGA-AD 0/1 at week 52 remained similar to those at week 16 across all baricitinib groups. NRS-4 improvement was maintained through week 52 with baricitinib 1mg and 2mg, but it was not statistically significant for baricitinib 4mg. Improvements in DLQI were greater with the baricitinib groups compared to placebo, but overall lower at week 52 than it was at week 16.

A phase 3, (RDBPC) study (BREEZE-AD PEDS) randomized participants aged 2 to < 18 years old (1:1:1:1) to baricitinib 1mg, 2mg, 4mg, and placebo daily for 16 weeks [50]. The results are as follows, respectively, for baricitinib 1mg, 2mg, 4mg, and placebo: vIGA-AD 18.2%, 25.8%, 41.7%, 16.4% (primary endpoint), with 4mg baricitinib being superior to placebo; EASI-75 32.2%, 40%, 52.5%, 32%; EASI-90 11.6%, 21.7%, 30%, placebo 12.3%; SCSORAD 75 7.4%, 15.8%, 20%, 9.8%; EASI change from baseline 15.67, 15.83, 16.88, 14.16; NRS ≥ 4 for itch 17.5%, 26.8%, 35.5%, 16.4%. Baricitinib 4mg had significant improvement for secondary endpoints at week 16 but baricitinib 1mg and 2mg did not.

The most common adverse events (≥ 5%) were nasopharyngitis, herpes simplex, headache, back pain in all groups, with baricitinib 4mg also experiencing conjunctivitis, diarrhea, upper abdominal pain, erysipelas, and urinary tract infection (UTI) in BREEZE-AD4. In BREEZE-AD PEDS, abdominal pain, acne, and headache were the most frequently reported AEs. Most adverse events were mild to moderate in severity, with serious AE most frequently occurring in the baricitinib 1mg and 4mg groups. There was one major adverse cardiovascular event in BREEZE-AD4 in the baricitinib 2mg group but was deemed to be unrelated to baricitinib. There were no venous thromboembolic events, gastrointestinal perforations, malignancies, cases of tuberculosis or confirmed opportunistic infections.

## Comparing Old and New Systemic Treatments

Drucker, et.al, performed a meta-analysis of 39 randomized clinical trials for systemic immunomodulatory treatments for patients with AD [51•]. The analysis included 20 medications and trials of 8 weeks or more, with most studies receiving up to 16 weeks of therapy. This study used the standardized mean difference (SMD) scale to compare dupilumab with older systemic AD medications as there are no head-to-head trials comparing the older systemic treatments for AD. The analysis showed that dupilumab and higher-dose cyclosporine had better improvements in clinical signs, itch, and QoL when compared to methotrexate and azathioprine. It also showed that Dupilumab 300mg q2w was superior to placebo for mean change in EASI with high certainty, while baricitinib 2mg qd, baricitinib 4mg qd, tralokinumab 150mg q2w, and tralokinumab 300mg q2w were superior to placebo with moderate certainty. Dupilumab 300mg q2w (high certainty), abrocitinib 100mg qd and 200mg qd (low certainty) were associated with clinically significant differences in DLQI scores compared with placebo. Dupilumab, abrocitinib 100mg and 200mg, upadacitinib 15mg and 30mg had clinically relevant improvements in the POEM score compared to placebo.

In an updated meta-analysis published in 2022, Drucker, et. al, added 21 new studies for a total of 60 trials [52•]. This update was the first trial with data comparing an oral JAK inhibitor (abrocitnib) against dupilumab directly, and the analysis showed that EASI, POEM, DLQI, PP-NRS were slightly more reduced (i.e. improved) with abrocitinib 200mg qd and upadacitinib 30mg qd when compared to dupilumab 300mg q2w. The outcomes were slightly less reduced with abrocitinib 100mg qd, baricitinib 2mg qd, tralokinumab 300mg q2w than dupilumab. Upadacitinib 15mg daily had similar effects as dupilumab. However, abrocitinib 100mg qd was associated with 2.6 times the odds of serious AE when compared with dupilumab but with 1.4 times the odds for abrocitinib 200mg qd. Additionally, there were lower rates of serious AE for dupilumab compared with placebo. SMD analysis had similar outcomes as the baseline meta-analysis (there were no new data for azathioprine, cyclosporine, or methotrexate), showing that high dose cyclosporine (300 mg/dy or 4–5 mg/kg/d) had improved clinical signs slightly better than dupilumab (low certainty). Lower dose cyclosporine (150 mg/d or < / = 3 mg/kg/d), methotrexate, and azathioprine had slightly less reduced signs than dupilumab with low certainty.

A systemic review and network meta-analysis by Chu et. al, studied 149 trials with 75 interventions for children and adults with moderate-to-severe AD [53•]. The analysis included trials that directly compared dupilumab vs abrocitinib or upadacitinib. High-dose upadacitinib was the most effective overall for five of six patient-important outcomes – in improving EASI, POEM, itch, DLQI, and reducing AD flares (Table 3).

**Table 3:** Comparison of Patient-Important Outcomes in Different Interventions

	EASI (%)	POEM	Itch Severity/ NRS	Sleep Disturbance	DLQI	Reducing AD flares	Highest Frequency of Any Adverse Event
High Efficacy	- High-dose Upadacitinib - High-dose Cyclosporine	- High-dose Upadacitinib	- High-dose Upadacitinib	- Dupilumab - High-dose Abrocitinib - High-dose Baricitinib - Lebrikizumab - Nemolizumab - High-dose Cyclosporine	- High- and low-dose Upadacitinib	- High- and low-dose Upadacitinib - High-dose Abrocitinib	- High-dose Upadacitinib - High-dose Cyclosporine
Moderate Efficacy	- Dupilumab - Low-dose Upadacitinib	- High-dose Abrocitinib - Dupilumab - Low-dose Upadacitinib	- High- & low-dose Abrocitinib - Dupilumab - Lebrikizumab - Nemolizumab - Low-dose Updacitinib		- Dupilumab - High-dose Abrocitinib - Lebrikizumab	- Low-dose Abrocitinib - Dupilumab - Tralokinumab	- Low-dose Upadacitinib -High-dose Abrocitinib -High-dose Baricitinib - Azathioprine -Low-dose Cyclosporine -Methotrexate
Not significantly different from Placebo	- Low dose Baricitinib - Azathioprine - Nemolizumab	- Low-dose Baricitinib - Nemolizumab - Omalizumab	- High- & low-dose Baricitinib - Low-dose cyclosporine - Tralokinumab	- Low-dose Abrocitinib - Low-dose Baricitinib - Tralokinumab	- Low-dose Abrocitinib - High- and low-dose Baricitinib - Nemolizumab - Tralokinumab		- Dupilumab - Tralokinumab -Low-dose Abrocitinib - Low-dose Baricitinib

High-dose upadacitinib was the most effective in improving EASI, when compared to placebo (high certainty). Dupilumab and low-dose upadacitinib had intermediate superior effectiveness (high certainty), and high-dose cyclosporine had superior effectiveness (low certainty). Low-dose (1-mg) baricitinib and azathioprine, nemolizumab, were not significantly different compared to placebo (moderate certainty). The recent Joint Task Force guidelines have recommended against the use of low-dose baricitinib [14••].

High-dose upadacitinib was the most effective in improving POEM, a measure of patient-reported AD severity (high certainty). High-dose abrocitinib, dupilumab, and low-dose upadacitinib had intermediate superior effectiveness (high certainty). Low-dose baricitinib, nemolizumab, and omalizumab were not clearly different from placebo (moderate or high-certainty).

Itch severity was measured by NRS, and high-dose upadacitinib was the most effective in improving NRS. High-dose and low-dose abrocitinib, dupilumab, lebrikizumab, nemolizumab, and low-dose upadacitinib had intermediate effectiveness (high certainty). High-dose and low-dose baricitinib, low-dose cyclosporine, and tralokinumab were not clearly different from placebo (moderate-high certainty).

Sleep disturbance was measured through NRS, and high-dose abrocitinib, high-dose baricitinib, dupilumab, lebrikizumab, nemolizumab, and narrow-band UVB were the most effective (moderate-to-high certainty), along with high-dose cyclosporine (low certainty). Low-dose abrocitinib, low-dose baricitinib, and tralokinumab were not clearly different from placebo (high certainty).

DLQI was used to measure QoL and high-dose and low-dose upadacitinib were among the most effective (high certainty). High-dose abrocitinib, dupilumab, and lebrikizumab were among those with intermediate superior effectiveness (high certainty). Low-dose abrocitinib, high- and low-dose baricitinib, nemolizumab, and tralokinumab were not clearly different from placebo (moderate or high certainty).

High-dose abrocitinib, high-dose and low-dose upadacitinib were among the most effective in reducing AD flares (high certainty). Low-dose abrocitinib, dupilumab, and tralokinumab were among the intermediate effectiveness (high certainty). There was low certainty evidence for oral corticosteroids and methotrexate in measuring the above outcomes.

High-dose upadacitinib had the highest frequency of any AE (high certainty). High-dose abrocitinib, high-dose baricitinib, low-dose upadacitinib had intermediate harm when assessed for frequency of any AE (high certainty). Dupilumab and tralokinumab were among the least harmful in any AE (high certainty) and in serious AE (moderate certainty). However, dupilumab, tralokinumab, and lebrikizumab were similarly harmful in having increased frequency of conjunctivitis (moderate-to-high certainty evidence). There was low/very low certainty regarding all other interventions.

High-dose upadacitinib and high-dose abrocitinib had the most frequent viral skin infections (moderate certainty), while dupilumab had a similar frequency of viral skin infections when compared to placebo (high certainty). Dupilumab and tralokinumab were the most protective against any skin infections (high certainty).

Although high-dose upadacitinib was the most effective for five of six outcomes, it had the highest frequency of any adverse event. Moderate certainty evidence demonstrated that dupilumab and tralokinumab had the least frequent serious adverse events (Table 3).

## Conclusion

In the past, immunosuppressants (DMARDs) and oral corticosteroids were commonly used to treat moderate-to-severe AD when topical treatments were insufficient. However, since 2017, various systemic treatments including dupilumab, tralokinumab, abrocitinib, upadacitinib and baricitinib (Europe Union) have been approved for patients with moderate-to-severe AD. While there are no head-to-head trials comparing the immunosuppressants with the newer treatment options, recent meta-analyses indicate that high-dose cyclosporine and dupilumab yield better AD outcomes compared to methotrexate and azathioprine. However, cyclosporine is not suitable for long-term use due to potential adverse effects like nephrotoxicity, hypertension, increased infection, and malignancy risks.

Clinicians are moving away from these older immunosuppressive medications, given the ongoing approval of newer immunomodulator treatments. These recent treatments have demonstrated efficacy in phase 3 trials. Dupilumab has been shown to be safe and effective in long-term phase 3 trials. The main side effects have been about 19% of conjunctivitis, most of which can be managed without stopping dupilumab. Long-term phase 3 trials with upadacitinib, abrocitinib and baricitinib showed that these medications have rapid onset of efficacy and anti-itch effects, which are maintained up to 48 to 52 weeks. However, these medications are associated with nausea, acne, and an increased risk of herpes zoster infection.

Tralokinumab has also been shown to be effective for patients with moderate-to-severe AD, as compared to placebo. In addition, when responders at 4 months switched from the recommended q2w regimen to q4w, the efficacy was maintained in most of these patients. Like dupilumab, tralokinumab is relatively safe with conjunctivitis as one of the most common AE (about 11%).

Direct comparison of dupilumab vs upadacitinib showed better efficacy and more rapid onset of anti-itch effect in upadacitinib. Direct comparison between dupilumab vs abrocitinib also showed more rapid onset of anti-itch effect in abrocitinib. The difference in anti-itch effect by abrocitinib was maintained through 26 weeks. However, the overall efficacy of dupilumab and abrocitinib is comparable. These comparisons are consistent with more recent meta-analyses which showed that high-dose upadacitinib is the more effective intervention, followed by dupilumab, high-dose abrocitinib, tralokinumab, high-dose cyclosporine and non-significant low-dose baricitinib. However, upadacitinib, abrocitinib, baricitinib and high-dose cyclosporine are associated with a higher rate of adverse events. While short-term controlled studies (12–16 weeks) showed no significant increase in the incidence of the major blackbox warnings: major cardiovascular events, thromboembolism, serious infections, and malignancy [54, 55], there were isolated events of these adverse effects in the long-term uncontrolled portion of the trials [47, 48, 56]. These events occurred mostly in patients who are 50 years and older, some of whom also had cardiovascular risk factors. These potential adverse events will need to be confirmed in longer term or post-marketing studies. In addition, any potential association with malignancy will also need longer term follow-up. Therefore, when deciding on oral JAK inhibitors for moderate-to-severe AD, age and risk factors for cardiovascular disease, thromboembolic events, serious infections, and malignancy will need to be considered.

Other patient-related issues include mode of administrations. Injection is associated with pain and may not be tolerated by some patients. On the other hand, oral JAK inhibitors will require regular blood monitoring. Patients with moderate-to-severe AD and co-morbidities such as asthma may benefit from dupilumab, whereas patients with concurrent alopecia may benefit from oral JAK inhibitors. Currently, FDA recommends biologics as the first-line systemic treatment for patients with moderate-to-severe AD. Patients on dupilumab who encounter adverse effects such as severe conjunctivitis may be considered for tralokinumab, given that a recent case series showed efficacy without recurrence of conjunctivitis [57]. In addition, older patients in whom oral JAK inhibitors are not advised, the efficacy of tralokinumab has also been shown [58]. On the other hand, patients who fail dupilumab due to an insufficient efficacy may be considered for oral JAK inhibitors, or tralokinumab (in older patients). Ongoing research includes alternative systemic treatments for AD, such as nemolizumab, rocatinlimab and amlitelimab.

## Data Availability

No datasets were generated or analysed during the current study.
